# The dilemma of selecting a first line CDK4/6 inhibitor for hormone receptor-positive/HER2-negative metastatic breast cancer

**DOI:** 10.1038/s41523-023-00520-7

**Published:** 2023-03-22

**Authors:** Albert Grinshpun, Sara M. Tolaney, Harold J. Burstein, Rinath Jeselsohn, Erica L. Mayer

**Affiliations:** 1 Medical Oncology, Dana-Farber Cancer Institute, Boston, MA USA; 2 Breast Oncology Program, Dana-Farber Brigham Cancer Center, Boston, MA USA; 3 Harvard Medical School, Boston, MA USA

**Keywords:** Breast cancer, Breast cancer

## Abstract

The combination of an endocrine agent with a CDK4/6 inhibitor is the standard of care in the first-line setting for patients with hormone receptor-positive, HER2-negative metastatic breast cancer. Randomized trials have demonstrated similar and significant improvements in progression-free survival using the three available CDK4/6 inhibitors and led to regulatory approval. However, mature overall survival data now suggest potential differences among the various agents, suggesting an evolution in selection preferences.

The development of cyclin dependent kinase 4/6 inhibitors (CDK4/6i) is one of the seminal advancements achieved in the last decade in the treatment of hormone receptor-positive HER2-negative (HR+/HER2−) metastatic breast cancer (MBC). CDK4/6i are usually administered with an endocrine backbone and lead to cell cycle arrest by targeting the cell cycle machinery^1^. Multiple large randomized trials have demonstrated substantial clinical benefit from the use of CDK4/6i in the first-line and pre-treated settings for metastatic HR +/HER2− disease^2^.

The three FDA approved agents in the metastatic setting are palbociclib, ribociclib and abemaciclib. Palbociclib was the first agent to be approved and has been the leading drug in the U.S. market, with ribociclib and abemaciclib comprising a smaller market share^3^. Overall, these drugs form the backbone of contemporary therapy, used in combination with endocrine therapy typically in the first-line setting for metastatic HR + disease^4^. All three CDK4/6i are orally bioavailable but have some differences in their chemical, biologic and pharmacologic features^2,4^. Palbociclib and ribociclib are given in an intermittent fashion and, while both have high selectivity for CDK4 and CDK6, ribociclib has a higher CDK4:CDK6 inhibition ratio (~4) given its weaker potency for inhibition of CDK6^5^. Abemaciclib has a different chemical structure and exhibits the highest inhibitory effect on CDK4/6 with a CDK4:CDK6 inhibition ratio of 5, and additional activity on multiple kinases^5^. Moreover, the agents exhibit different acquired resistance mechanisms, demonstrated in a recent high-resolution analysis of pre-clinical models, providing further support for their disparate nature^6^. Clinically, abemaciclib is given continuously, has proven blood-brain barrier penetration and is approved as monotherapy in pre-treated patients^7^. Despite substantial translational work across the large pivotal metastatic trials, currently the only predictive biomarker for CDK4/6 benefit is estrogen receptor positivity (ER+). In the adjuvant setting, as supported by the MONARCH-E study, the addition of 2 years of abemaciclib to adjuvant endocrine therapy improves disease-free survival, a finding which led to FDA approval for high-risk node positive HR + disease in October 2021^5,8^. An update at 4 years of follow-up has demonstrated continued and further improvement in invasive disease-free survival between the treatment and control groups, implying a carry-over effect beyond the active treatment period^9^. Evaluation of the addition of palbociclib to endocrine therapy in the adjuvant setting has been explored in the PALLAS and PENELOPE-B trials but did not demonstrate benefit beyond endocrine therapy alone^10,11^. The NATALEE trial (NCT03701334) of adjuvant ribociclib has completed accrual; however, the results are pending. Toxicity profiles differ among these three agents. Abemaciclib causes predominantly gastrointestinal toxicity, whereas palbociclib and ribociclib are characterized by hematologic toxicity, notably asymptomatic neutropenia. In contrast to palbociclib, both abemaciclib and ribociclib require liver function monitoring. Also, ribociclib may prolong the QTc interval in a small proportion of patients (<5%), necessitating ECG monitoring during the first 2 cycles^2,12^.

During the last decade, 7 pivotal prospective randomized controlled trials have established the roles of palbociclib, ribociclib and abemaciclib combined with an endocrine partner in the treatment of first-line (Table 1) and pre-treated patients with HR + MBC. Despite several nuanced distinctions in study design including differences in study populations, the hazard ratios for progression-free survival (PFS) benefit are strikingly similar across trials and in the range of 0.5, regardless of prior treatment exposures^13–19^. However, as overall survival (OS) results have matured and been reported, apparent differences among the studies have emerged. In pre-treated patients with MBC, the regimens of fulvestrant combined with either ribociclib or abemaciclib have demonstrated improved OS, compared to fulvestrant alone^20–22^. Palbociclib combined with fulvestrant did not reach statistical significance in the intention-to-treat (ITT) population, achieving an OS benefit only in the subset of patients with endocrine sensitive disease; however, this trial included patients who had received significantly more prior therapies in the metastatic setting compared to patients on the MONARCH-2 and MONALEESA-3 trials, investigating abemaciclib and ribociclib, respectively, in the pre-treated setting^23^.

**Table 1:** Results from pivotal prospective randomized controlled trials investigating palbociclib, ribociclib and abemaciclib combined with an endocrine partner in the treatment of first-line patients with HR + MBC.

Trial Details	PALOMA-2^13,26^	MONALEESA-2^12,15^	MONALEESA-3^20,22,24^	MONALEESA-7^18,25^	MONARCH-3^14,29^
CDK4/6 inhibitor	Palbociclib	Ribociclib	Ribociclib	Ribociclib	Abemaciclib
Endocrine therapy	Letrozole	Letrozole	Fulvestrant	Goserelin plus tamoxifen or NSAI	NSAI
Menopausal status	Post-menopausal	Post-menopausal	Post-menopausal	Pre/peri-menopausal	Post-menopausal
Sample size	666	668	365 (1L therapy)	672	493
Median follow up (months)	90	80	70.8	53.5	70.2
mPFS combination arm (months)	24.8	25.3	33.6	23.8	28.2
mPFS control arm (months)	14.5	16	19.2	13	14.8
mPFS HR (95% CI)	0.58 (0.46–0.72)	0.57 (0.46–0.7)	0.55 (0.42–0.72)	0.55 (0.44–0.69)	0.54 (0.42–0.69)
mPFS *p* value	<0.001	<0.001	n/a	<0.001	<0.001
mOS combination arm (months)	53.9	63.9	67.6	58.7	67.1
mOS control arm (months)	51.2	51.4	51.8	48	54.5
mOS HR (95% CI)	0.956 (0.777–1.177)	0.76 (0.63–0.93)	0.67 (0.5–0.9)	0.76 (0.61–0.96)	0.754 (0.584–0.974)
mOS *p* value	0.3378	<0.001	n/a	<0.001	0.0301^a^
% ≥1 dose reduction due to AE (combination arm)	36%	50.6%	n/a	31%	46.5%
% treatment discontinuation (combination arm)	9.7%	7.5%	n/a	4%	16.5%

*1L* first-line, *NSAI* non-steroidal aromatase inhibitor, *mPFS* median progression-free survival, *mOS* median overall survival, *AE* adverse events.

^a^Results from preplanned interim analysis, the statistical significance threshold for abemaciclib superiority was not met.

OS data, a secondary endpoint of the first-line metastatic trials, have been presented in final analyses for ribociclib and palbociclib, and as a preplanned interim analysis for abemaciclib. In the MONALEESA-2 trial, at a median follow-up of 6.6 years, the combination of ribociclib with letrozole demonstrated a significant improvement in OS over letrozole alone, with a median OS of 64 months in the treatment arm, compared with 51 months in the control arm (HR 0.76; 95% CI, 0.63–0.93; two-sided *P* = 0.008)^12^. Similarly, for patients treated with ribociclib and fulvestrant as their first-line treatment in the MONALEESA-3 trial, the combination showed a statistically significant advantage versus fulvestrant alone; at a median follow-up of 70.8 months, the median OS was 68 months in the combination arm versus 52 months in the fulvestrant alone arm (HR 0.67; 95% CI, 0.5–0.9)^24^. Furthermore, focusing on pre-/peri-menopausal women with HR + MBC, an exploratory analysis of the MONALEESA-7 trial with 54 months of follow-up reported a significant improvement in OS with the addition of ribociclib to endocrine therapy versus placebo, with a median OS of 59 months with use of the CDK4/6i compared to 48 months in the control arm (HR 0.76; 95% CI, 0.61–0.96)^18,25^. In the PALOMA-2 trial, however, the combination of palbociclib with letrozole in the first-line setting did not demonstrate an OS benefit^26^. At 7.5 years follow up, the median OS of palbociclib and letrozole in the ITT population was 54 months, not significantly longer than 51 months in the control arm of endocrine therapy alone (HR 0.956; 95% CI, 0.777–1.177; 1-sided *P* = 0.3378). An important finding in this updated PALOMA-2 report was the notable amount of missing survival data, 13% in the palbociclib arm and 21% in the placebo arm, due to patients who were lost to follow-up or censored. For those patients for whom post-progression therapy was reported, a greater percentage in the control arm received CDK4/6i than the palbociclib arm (27% versus 12%, the majority being palbociclib). A sensitivity analysis was performed to adjust for patients with missing data yet did not reach statistical significance for OS. Of note, 10% of patients on the study arm continue on palbociclib, at 7.5 years of follow-up. In comparison to the PALOMA-2 report, real-world data analyses of U.S.-based outcomes for patients receiving first-line endocrine based therapy have reported PFS and OS benefits for the combination of palbociclib and letrozole over letrozole alone, with an observed improvement in median PFS from 11.9 months to 20.0 months (HR 0.58; 95% CI 0.49–0.69; *P* < 0.0001), and median OS from 40.4 to 53.4 months (HR 0.67; 95% CI, 0.60–0.76; *P* < 0.0001)^27,28^. As for abemaciclib in the first-line setting, in a pre-specified interim survival analysis of the MONARCH-3 trial at a median follow up of 70.2 months, the addition of abemaciclib to NSAI demonstrated a median OS of 67.1 months compared to 54.5 months for placebo plus NSAI (HR = 0.754, 95% CI, 0.584–0.974, two-sided *P* = 0.0301)^29^. Notably, the statistical significance threshold for abemaciclib superiority was not met, and further follow-up of the trial is ongoing. Interestingly, accounting for differences in duration of study follow-up, OS in the control endocrine arms in these trials is fairly consistent.

Given these results, how can we explain the disparate findings from the recent OS reports? With the remarkable similarity in PFS benefit reported across all of the HR + MBC CDK4/6i trials, what subsequent events and therapies in the long period of post-progression survival occurred for these patients? Are these findings related to actual differences in drugs, differences in trials, differences in patient population, or other features? And what do these findings mean for the care of patients with this disease?

There are several possible explanations for the observed difference in OS benefit between the PALOMA-2 and MONALEESA-2 trials^12,26^. To start, palbociclib received regulatory approval during the conduct of these studies, and as both trials were blinded and did not allow crossover, a considerable number of patients withdrew consent, possibly to receive commercial CDK4/6i outside of the clinical trial. The percent of patients with missing survival data was higher in PALOMA-2 than MONALEESA-2 (specifically in the placebo arm; 21% versus 9.9%, respectively). With an unknown complete crossover rate, an unequal effect on survival data cannot be ruled out. Furthermore, the post-progression systemic therapy exposure was different between these two trials. In MONALEESA-2, a higher percentage of patients in both arms were exposed to subsequent CDK4/6i; 22% in the ribociclib arm and 34% in the letrozole alone arm, compared with 12% of patients in the palbociclib arm and 27% in letrozole alone arm in PALOMA-2. Most patients (>75%) in both studies received palbociclib as the CDK4/6i of choice in the post-progression setting. The recently presented randomized phase II PACE trial provides useful information on the interpretation of the value of post-progression palbociclib^30^. In this trial, 220 patients with prior progression on CDK4/6i (90.9% prior palbociclib) were randomized to one of three arms: fulvestrant alone; palbociclib and fulvestrant; palbociclib, fulvestrant, and avelumab (PD-L1 inhibitor). Results demonstrated that the addition of palbociclib to fulvestrant beyond progression on previous CDK4/6i therapy did not improve PFS compared to fulvestrant alone. These results are in contrast to the phase II MAINTAIN study, which randomized patients with prior progression on CDK4/6i (87% of them treated with prior palbociclib) to placebo or ribociclib with a different endocrine backbone, and reported a significant PFS improvement when ribociclib was added^31^. Taken together, it is possible that switching from one CDK4/6i to another may add benefit, versus re-exposure to the same drug. For the phase III trials, subsequent CDK4/6i exposure may have not only improved outcomes for patients in the control arms, but also provided extra benefit only for the treatment arm in MONALEESA-2, while patients in the palbociclib arm in PALOMA-2 did not derive benefit from continuation of the same agent at progression. Additional studies of CDK4/6 post-progression are ongoing (e.g., postMONARCH [NCT05169567]) and results are pending. Subsequent post-progression treatment strategies in the MONALEESA-2 and PALOMA-2 trials are not known and may have been influenced by patterns of care in the countries from which patients were accrued.

In addition, although both trials enrolled patients globally, the actual population of accrued patients differed. The prevalence of Asian patients was distinct between the 2 trials; in MONALEESA-2, 7.6% of the patients were Asian, compared with 14.3% of patients enrolled in PALOMA-2. Asian patients may experience greater toxicity from both palbociclib and ribociclib treatment due to slower drug clearance^32,33^. Hypothetically, these differences in drug tolerability may have impacted OS outcomes.

It has been proposed that there may have been differences in endocrine sensitivity in the patient populations between the two trials, despite both trials enrolling a similar fraction of patients with de-novo MBC (about one-third). In MONALEESA-2, 60% of the patient population had a reported disease-free interval (DFI) longer than 2 years, suggesting endocrine sensitive disease. In PALOMA-2, 40% of patients were reported to have a DFI longer than 1 year, suggesting a potentially lower prevalence of patients with endocrine sensitivity.

In addition, PALOMA-2 reported 22% of the trial population had a DFI < 12 months, compared to 1% in MONALEESA-2^13,15^. However, it is important to note that different definitions of DFI and treatment-free interval (TFI) were used in the two studies. In MONALEESA-2, DFI was defined as the time from initial diagnosis to first recurrence, whereas in PALOMA-2, DFI was defined as the time from end of neo/adjuvant therapy to recurrence (similar to TFI)^15,34^. When applying a consistent definition of DFI < 12 months for both trial populations, fairly similar percentages of patients are identified with a short duration from end of adjuvant systemic treatment to disease recurrence (PALOMA-2; 22%, MONALEESA-2: 18%). In addition, the consistency in PFS and OS observed in the control arms in PALOMA-2 and MONALEESA-2 further supports the overall similarity of patient populations across these trials. This clarification in definition of endocrine sensitivity suggests that differences between the trials may not be due to substantial differences in the characteristics of the trial populations.

Finally, results from these studies may be related to the studies’ statistical design. Prior to the reported results in 2022, independent statistical estimates based on the intrinsic designs of the PALOMA-2 and MONALEESA-2 trials suggest statistical power <70% to demonstrate OS benefit of ≤12 months. The authors predicted that future discrepancies in reported outcomes are possible and may be attributed to chance rather than actual differences in drug efficacy^35^.

In summary, despite the similar chemical structures, appreciable pharmacological differences and identical primary endpoint PFS results, first-line trials using the CDK4/6i ribociclib and palbociclib, including MONALEESA-2 and PALOMA-2, reported different OS results, a finding which could be due to a number of reasons. Cross trial comparisons are not appropriate and are discouraged, as reasons for observed difference are likely multifactorial and cannot be unequivocally determined, however, these disparate results may truly reflect efficacy differences between the drugs. While there is no current randomized data comparing these agents, a unique ongoing trial, HARMONIA (NCT05207709), will randomize first-line patients with metastatic HR + HER2-enriched breast cancer to receive endocrine therapy with either palbociclib or ribociclib. This trial will generate the first head-to-head comparison of these agents, however, the data may only be applicable to patients with the specific intrinsic subtype of disease.

A significant question for breast cancer clinicians is how the current data landscape for CDK4/6i impacts contemporary patient care. The following guidance reflects consideration of the currently available data as well as internal discussions throughout our large academic practice. However, the future addition of new evidence may necessitate re-evaluation of this collective opinion.

When considering patients who have already been started on a palbociclib-containing regimen, it is important to remember that the combination of letrozole and palbociclib is an active regimen and that there are patients who continue to maintain disease stability many years after starting palbociclib at time of FDA approval in 2015. For patients who are currently stable on a palbociclib-based regimen without intolerable toxicity, it is recommended to maintain the current regimen and not change agents. However, for a newly diagnosed patient with metastatic ER+/HER2− breast cancer, the recent data likely will change practice patterns. Given the OS benefit in MONALEESA-2, endocrine therapy in combination with ribociclib could be prioritized when choosing first-line therapy, unless the patient has a potential contraindication such as pre-existing cardiac dysfunction, electrolyte imbalances, or liver disease. Recommendations for abemaciclib will be pending mature OS results of MONARCH-3.

Overall, the introduction of CDK4/6i into clinical practice has substantially improved care for patients with metastatic ER+/HER2− breast cancer, yet now is the time to refine and optimize our practice. The recent OS data have helped improve our understanding of the roles of the respective agents and confirms their utilization in the first-line metastatic setting. In addition, trials designed to overcome CDK4/6i-resistance exploring use of CDK4/6i post-progression are ongoing and will evaluate not only the continuation of CDK4/6i but also the introduction of other novel agents. More data from these studies will shed light on the potential differential efficacy of the CDK4/6i, their optimal sequencing, and might assist in enhanced future decision-making for the many patients with ER+/HER2− MBC.
